# Variation in bovine leptin gene affects milk fatty acid composition in New Zealand Holstein Friesian  ×  Jersey dairy cows

**DOI:** 10.5194/aab-64-245-2021

**Published:** 2021-06-07

**Authors:** Ishaku Lemu Haruna, Huitong Zhou, Jon G. H. Hickford

**Affiliations:** Faculty of Agriculture and Life Sciences, Lincoln University, Lincoln 7647, New Zealand

## Abstract

Leptin is a protein hormone secreted from white adipose tissue. It regulates food/feed intake, body weight, immune function and reproduction. In our investigation, the polymerase chain reaction (PCR) amplification coupled with single-strand conformational polymorphism (SSCP) analysis was used to reveal variation in bovine leptin gene (*LEP*) in New Zealand (NZ) Holstein Friesian × Jersey (HF × J) dairy cows. Subsequent sequence analysis of a 430 bp amplicon spanning the entirety of exon 3 and part of the intron 2 region revealed three variant sequences ($A_{3}$, $B_{3}$ and $C_{3}$) containing a total of five nucleotide substitutions, all of which have been reported previously. Using general linear mixed-effect model analyses, the presence of variant $A_{3}$ (the most common variant) was associated with a decreased level of C15:1, C18:1 *trans*-11, C18:1 all *trans*, C18:2 *trans*-9, *cis*-12, C22:0 and C24:0 levels but increased levels of C12:1 and C13:0 *iso* (p<0.05). Variant $B_{3}$ was associated with reduced levels of C6:0, C8:0, C11:0, C13:0 and C20:0 but increased C17:0 *iso* and C24:0 levels (p<0.05). Variant $C_{3}$ was associated with decreased C17:0 *iso* levels but increased C20:0 (p<0.05) levels. In a genotype model, the $A_{3}B_{3}$ genotype was associated with increased levels of C22:0 and C24:0 but decreased C8:0, C10:0, C11:0, C13:0, C15:0 and grouped medium-chain fatty acid (MCFA) levels (p<0.05). Genotype $A_{3}C_{3}$ was found to be associated with decreased levels of C10:0, C11:0, C13:0 and grouped MCFA (p<0.05). This is the first report of findings of this kind in NZ HF × J cows, and they suggest that variation in exon 3 of bovine leptin gene could be explored as a means of decreasing the concentration of saturated fatty acids in milk.

## 1 Introduction

There has been a growing interest in genomic selection programmes aimed at modifying the composition of milk fatty acids (FAs) using candidate gene approaches. In this respect, several genes have been implicated in affecting milk FA composition, including the leptin gene (*LEP*).

Bovine *LEP*, previously known as *OB*, *OBS* and *LEPD*, has been mapped to chromosome 4 (Pomp et al., 1997) and it encodes the protein leptin. This protein is secreted from white adipose tissue and has been found to regulate feed intake, energy partitioning and metabolism (Liefers et al., 2002; Lagonigro et al., 2003), as well as lactogenesis (Feuermann et al., 2004).

The hypothalamus is identified as the main site of leptin's activity in regulating food intake and energy expenditure. Leptin signals are converted into neural responses, and this results in changes in feed intake (Tang-Christensen et al., 1999). A neurotransmitter identified as neuropeptide Y (NPY) is associated with the regulation of food intake, and leptin exerts its effect by either stimulating or inhibiting the release of NPY. Among other things, this eventually results in a decrease in feed intake and an increase in energy expenditure (Houseknecht et al., 1998). There are also suggestions that leptin could also regulate fat mobilization (Halaas et al., 1995).

Previous reports have highlighted the effects of leptin gene variation on some livestock traits of economic value, such as the yield and quality of meat and milk obtained from farmed animals. For example, in sheep an effect of leptin gene variation on weaning weight was observed (Hajihosseinlo et al., 2012), while in cattle, leptin or leptin receptor gene polymorphisms have been associated with carcass FA composition (Kawaguchi et al., 2017), milk fat levels (Giblin et al., 2010; De Matteis et al., 2012) and milk FA composition (Pegolo et al., 2016).

Although the effects of bovine leptin variation on milk fat composition have been described in studies of other cattle breeds, so far there is no report of the effects of leptin gene variation on the composition of milk FA content or profile in New Zealand (NZ) Holstein Friesian × Jersey (HF × J) dairy cows that are permanently grazed outdoors on pasture. The aim of this study was therefore to investigate whether variation in the gene affected milk fat traits in these cows.

## 2 Materials and methods

### 2.1 The NZ dairy cattle investigated

This study was approved by the Lincoln University Animal Ethics Committee (AEC) under the provisions of the NZ Government's Animal Welfare Act 1999. A total of 300 NZ HF × J dairy cows (alternatively known as KiwiCross™ cows) of variable and unknown breed proportion and of 3 to 9 years of age were used in this investigation. These cows were from two herds, and all of them were grazed outdoors on pasture (a mixture of perennial ryegrass and white clover) on the Lincoln University Dairy Farm (LUDF; Canterbury, NZ). All the cows calved over the months of August–September and they were milked twice a day, in the morning and then in the afternoon.

### 2.2 Collection of milk samples for fatty acid analysis

The collection of milk samples from cows for FA analysis was carried out when they were 148 ± 19 d in milk (DIM) and in a single afternoon milking in mid-January. These samples were frozen at a temperature of -20${}^{\circ }$C, and then freeze-dried, before being individually ground to a fine powder for component analysis.

### 2.3 Gas chromatography of milk fatty acids

Prior to being analysed by gas chromatography (GC) as FA methyl esters (FAMEs), the milk FAs were methylated and then extracted in n-heptane. The methylation reactions were performed in 10 mL Kimax tubes. Individual freeze-dried and powdered milk samples (0.17 g) were dissolved in 900 µL of n-heptane (100 %, AR grade), before 100 µL of internal standard (5 mg/mL of C21:0 methyl ester in n-heptane) and 4.0 mL of 0.5 M NaOH (in 100 % anhydrous methanol) were added.

The tubes were vortexed prior to incubation in a block heater (Ratek Instruments, Australia) at 50${}^{\circ }$C for 15 min. After cooling to room temperature, another 2.0 mL of n-heptane and 2.0 mL of deionized water were added to each of the tubes. After vortexing, the tubes were centrifuged (Megafuge 1.0R, Heraeus, Germany) for 5 min at 1500× g. The top layer of n-heptane was transferred into a second Kimax tube, and 2.0 mL of n-heptane was added to each of the original tubes. The extraction was repeated, and the n-heptane aspirates were then pooled. Anhydrous sodium sulfate (10 mg) was added to the n-heptane extracts, to remove any residual water.

The GC analysis for milk FAs was carried out using a Shimadzu GC-2010 gas chromatograph (Shimadzu Corporation, Kyoto, Japan) equipped with a flame ionization detector and an AOC-20i autosampler. The output was analysed with GC Solution Software (Shimadzu). The analysis was carried out by injecting 1 µL of the n-heptane sample extract into a 100 m GC capillary column (250 µm × 0.25 µm, CP-Select, Varian) with a 1:60 split ratio. The separation was undertaken with a helium carrier gas, and it was run for 92 min. The temperature of both the injector and detector were set at 250${}^{\circ }$C, and the thermal profile of the column incubation consisted of 45${}^{\circ }$C for 4 min, followed by 27 min at 175${}^{\circ }$C (ramped at 13${}^{\circ }$C/min), 35 min at 215${}^{\circ }$C (ramped at 4${}^{\circ }$C/min), and a final temperature of 250${}^{\circ }$C for 5 min (ramped at 25${}^{\circ }$C/min).

The individual FAMEs were identified by comparing their peak retention times to commercially obtained external standards (ME61, ME93, BR3, BR2, ME100, GLC411 and GLC463; Larodan AB, Sweden). Quantification of the individual FAMEs was based on peak area assessment and comparison with the internal and external standards. The threshold for peak area determination on the chromatogram was a 500-unit count, and peaks under this threshold were ignored. The calculated minimum level of an individual FAME that could be identified was therefore 0.01 g per 100 g of total FA. After the FAs were individually measured, they were sorted into various groups and indices. These groups were the following: short-chain FAs (SCFAs) = C4:0 + C6:0 + C8:0; medium-chain FAs (MCFA) = C10:0 + C12:0 + C14:0; long-chain FAs (LCFAs) = C15:0 + C16:0 + C17:0 + C18:0 + C19:0 + C20:0 + C22:0 + C24:0; omega-3 FAs = C18:3 *cis*-9, 12, 15 + C20:5 *cis*-5, 8, 11, 14, 17 + C22:5 *cis*-7, 10, 13, 16, 19; omega-6 FAs = C18:2 *cis*-9, 12 + C18:3 *cis*-6, 9, 12 + C20:3 *cis*-8, 11, 14 + C20:4 *cis*-5, 8, 11, 14; monounsaturated FAs (MUFA) = C10:1 + C12:1 + C14:1 *cis*-9 + C15:1 + C16:1 *cis*-9 + C17:1 + C18:1 *trans*-11 + C18:1 *cis*-9 + C18:1 *cis*-(10 to 15) + C20:1 *cis*-5 + C20:1 *cis*-9 + C20:1 *cis*-11 + C22:1 *trans*-13; polyunsaturated FAs (PUFA) = C18:2 *trans*-9, 12 + C18:2 *cis*-9, *trans-*13 + C18:2 *cis*-9, *trans*-12 + C18:2 *trans*-9, *cis*-12 + C18:2 *cis*-9, 12 + C18:3 *cis*-6, 9, 12 + C18:3 *cis*-9, 12, 15 + conjugated linoleic acid (CLA) + C20:3 *cis*-8, 11, 14 + C20:4 *cis*-5, 8, 11, 14 + C20:5 *cis*-5, 8, 11, 14, 17 + C22:5 *cis*-7, 10, 13, 16, 19; and total branched FA = C13:0 *iso* + C13:0 *anteiso* + C15:0 *iso* + C15:0 *anteiso* + C17:0 *iso*.

Unsaturated FA indices were also calculated as follows: C12:1 index (C12:1 divided by the sum of C12:0 and C12:1); C14:1 index (C14:1 *cis*-9 divided by the sum of C14:0 and C14:1 *cis*-9); C16:1 index (C16:1 *cis*-9 divided by the sum of C16:0 and C16:1 *cis*-9) and C18:1 index (C18:1 *cis*-9 divided by the sum of C18:0 and C18:1 *cis*-9). The method is as described by Li et al. (2019), with the un-adjusted mean levels in the 300 cows being calculated and used subsequently in the statistical analyses.

### 2.4 Blood sample collection

Using either the piercing of the animal's ear or the tail vein (as approved under the Code of Welfare, section 75 and 76 of the NZ Animal Welfare Act 1999), blood samples were collected from each cow onto FTA™ cards (Whatman™, Middlesex, UK). The samples were air-dried and DNA purification was carried out using a two-step procedure described by Zhou et al. (2006).

### 2.5 Amplification with the polymerase chain reaction (PCR)

Using the following forward and reverse primers (5${}^{\prime }$-TTGCTCTCCCCTTCCTCCTG-3${}^{\prime }$ and 5${}^{\prime }$-CTCAGGTTTCTTCCCTGGAC-3${}^{\prime }$ respectively) adapted from the work of Haruna et al. (2020), the entirety of exon 3 and part of the intron 2 region of the bovine leptin gene was amplified. This region was selected for investigation because it is highly polymorphic in comparison to the exon 2 region, and previous report has revealed associations of exon 3 with FA composition in muscle (Orrù et al., 2011). The PCR reactions were undertaken in 15 µL volumes containing the genomic DNA on a 1.2 mm diameter disc of the FTA™ card, 0.25 µM for each primer, 150 µM for each dNTP (Eppendorf, Hamburg, Germany), 3.0 mM Mg${}^{2+}$, 0.5 U of *Taq* DNA polymerase (Qiagen, Hilden, Germany), and 1× the reaction buffer supplied with the enzyme.

The amplification was carried out in Bio-Rad S1000 thermal cyclers (Bio-Rad, Hercules, CA, USA). The thermal cycling conditions included an initial denaturation at 94 ${}^{\circ }$C for 2 min, followed by 35 cycles of 94 ${}^{\circ }$C for 30 s, annealing for 30 s at 60 ${}^{\circ }$C, extension at 72 ${}^{\circ }$C for 30 s and a final extension step at 72 ${}^{\circ }$C for 5 min.

### 2.6 Single-strand conformational polymorphism (SSCP) analyses

An SSCP technique was used to detect genetic variation in the amplicons obtained from the PCR reactions. The choice of SSCP was because it is inexpensive and can screen for variation in a large number of cattle breeds, thus giving a better representation of the entire breed. Also, it is a reliable, reproducible and effective analytical method for the detection of deletions, insertions or rearrangement in PCR-amplified DNA sequence. Briefly, following PCR amplification, a 0.7 µL aliquot of the PCR reactions was added to 7 µL of loading dye containing 10 mM ethylenediaminetetraacetic acid (EDTA), 0.025 % bromophenol blue, 0.025 % xylene cyanol, and 98 % formamide. The samples were then placed on a hot plate already set at 95 ${}^{\circ }$C, for 5 min to enable DNA denaturation. This was followed by snap chilling on wet ice. Samples were then loaded onto 16 cm × 18 cm, 10 % acrylamide : bisacrylamide (37.5:1) (Bio-Rad) gels containing 4 % glycerol. Electrophoresis was carried out using Protean II xi cells (Bio-Rad) for 24 h at 390 V and 15 ${}^{\circ }$C in 0.5× Tris/Borate/EDTA running buffer.

To detect the SSCP banding patterns, the gels were silver-stained using a method described by Byun et al. (2009).

### 2.7 Nucleotide sequencing

Based on the PCR-SSCP patterns observed, cattle that were homozygous with unique banding patterns were sequenced directly. For heterozygous variants, the unique band(s) was excised from the wet gel, incubated in water at 69 ${}^{\circ }$C for 1 h, and subsequently amplified and sequenced based on the approach described by Gong et al. (2011). The sequences were then aligned, and other analyses were undertaken using DNAMAN (Version 5.2.10, Lynnon Biosoft, Vaudreuil, Canada) to enable identification of the position of the nucleotide variation.

### 2.8 Statistical analysis

The statistics software IBM SPSS version 22 (IBM, Armonk, NY, USA) was used to perform all statistical analyses, and an alpha level of p<0.05 was set as a threshold for acceptance of association.

The age of the cow expressed in an integer value of years (i.e. as a categorical variable in a range from 3 to 9 years of age), the number of days in milk for each cow (DIM; expressed as an integer value but entered into the model as a continuous trait) and herd (to correct for herd-specific effects) were fitted to the models as fixed explanatory factors.

Using general linear mixed-effects models (GLMMs), associations between *LEP* variants and variation in milk FA component levels were tested.

First, single-variant presence/absence models (each variant was coded as present (1) or absent (0) for each animal's genotype) were used to ascertain which variant(s) should be analysed in subsequent multi-variant models. The multi-variant models included any variant that had a variant-FA trait association in the single-variant presence/absence analysis with a p value of less than 0.200. This is a low threshold for the inclusion of a possibly explanatory factor in the model. The multi-variant models were also corrected for the other factors described above.

For genotypes with a frequency greater than 5 % (thus having adequate sample size per group), the effect of variation in a cow's *LEP* genotype on the component levels of individual and grouped FAs was tested using general linear mixed-effects models (GLMMs) and multiple pair-wise comparisons (least significant difference tests) with Bonferroni corrections.

The model was Y${}_{ijkl}=$ μ + $G_{i}+$ $A_{j}+$ $D_{k}+$ $H_{l}+$ $e_{ijkl}$ for the genotype, where Y${}_{ijkl}$ is the observed trait value in the ijklth cow; μ is the mean trait value for a given trait; $G_{i}$ is the fixed effect of ith *LEP* genotype; $A_{j}$ is the effect of age (j=3–9 years); $D_{k}$ the effect of the number of days the cow has produced milk (DIM: k=94–186 d); $H_{l}$ the fixed effect of lth farm (l=1 or 2); and $e_{ijkl}$ is the random error.

The effect of sire of cow could not be included in the GLMMs, because some semen straws (sire genetics) used in NZ dairy cattle artificial insemination-based breeding approaches contain mixed-sire semen purchased from commercial semen producers. In these cases, it is impossible to ascertain individual sire identity. However, since the straws were mixed-semen straws and because different sires are used for different inseminations, in different years, it is unlikely that sire was a strongly confounding effect. Cow age and herd might also be confounded with sire, but this cannot be confirmed.

## 3 Results

### 3.1 SNPs identified in the bovine leptin gene

**Figure 1:**
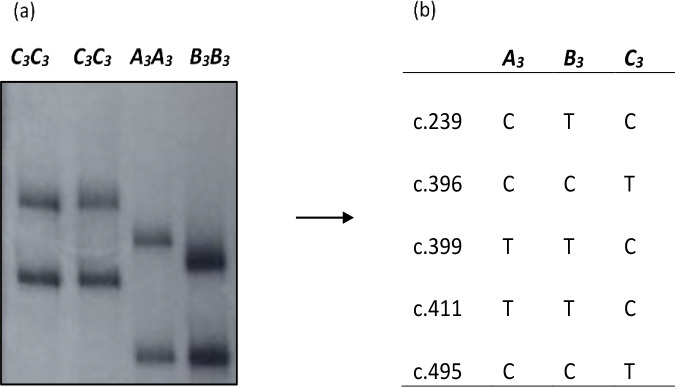
**(a)** PCR-SSCP banding patterns obtained in the exon 3/intron 2 region of bovine leptin gene investigated. **(b)** Nucleotide sequencing revealed the different nucleotide sequence variations identified in the region investigated.

Using the primers 5${}^{\prime }$-TTGCTCTCCCCTTCCTCCTG-3${}^{\prime }$ and 5${}^{\prime }$-CTCAGGTTTCTTCCCTGGAC-3${}^{\prime }$, a fragment of approximately 430 bp length and consisting of the entire exon 3 and part of intron 2 region of bovine leptin gene was amplified and analysed using the PCR-SSCP analyses. The PCR-SSCP analyses coupled with DNA sequencing revealed three banding patterns ($A_{3}$, $B_{3}$ and $C_{3}$) with NCBI GenBank accession numbers MN119553, MN119554 and MN119555 respectively in the region investigated (Fig. 1). A total of five single-nucleotide substitutions – c.239C/T (p.Ala80Val), c.396C/T (p.Gly132=), c.399T/C (p.Val133=), c.411T/C (p.Ala137=) and c.495C/T (p.Pro165=) in exon 3 – were identified, all of which have been reported previously (Haruna et al., 2020).

### 3.2 Variant presence/absence models

The results of the general linear mixed effect models revealed that the presence (or absence) of variants $A_{3}$, $B_{3}$ and $C_{3}$ in a cow's genotype was associated with the quantity of some milk FA methyl esters (FAMEs), with different variants having different effects as detailed in Table 1. The presence of variant $A_{3}$ (the most common variant) was associated with decreased C15:1, C18:1 *trans*-11, C18:1 all *trans*, C18:2 *trans*-9, *cis*-12, C22:0 and C24:0 levels but increased levels of C12:1 and C13:0 *iso* (p<0.05). Variant $B_{3}$ was revealed to be associated with reduced levels of C6:0, C8:0, C11:0, C13:0 and C20:0 but increased C17:0 *iso* and C24:0 levels (p<0.05). Variant $C_{3}$ was associated with decreased C17:0 *iso* level but an increased level of C20:0 (p<0.05).

**Table 1:** Associations between bovine leptin gene variants with average quantity of individual and grouped milk fatty acid methyl ester (FAME) in New Zealand (NZ) HF × J cows.

	Mean ± SE${}^{1}$ (g/100 g milk FA)						
Individual/	Variants	Other	Absent	n	Present	n	p
grouped		variants					
fatty acids${}^{2}$		in model					
C4:0	$A_{3}$	none	1.28 ± 0.035	14	1.27 ± 0.010	286	0.760
	$B_{3}$	none	1.27 ± 0.013	123	1.26 ± 0.012	177	0.583
	$C_{3}$	none	1.26 ± 0.010	247	1.28 ± 0.018	53	0.482
C6:0	$A_{3}$	none	1.56 ± 0.032	14	1.56 ± 0.009	286	0.871
	$B_{3}$	none	1.57 ± 0.011	123	1.55 ± 0.010	177	**0.038**
	$C_{3}$	none	1.56 ± 0.009	247	1.56 ± 0.016	53	0.955
C8:0	$A_{3}$	none	1.17 ± 0.026	14	1.18 ± 0.007	286	0.598
	$B_{3}$	none	1.19 ± 0.010	123	1.17 ± 0.009	177	**0.048**
	$C_{3}$	none	1.18 ± 0.008	247	1.17 ± 0.014	53	0.450
C10:0	$A_{3}$	none	3.12 ± 0.100	14	3.25 ± 0.028	286	*0.193*
	$B_{3}$	none	3.28 ± 0.036	123	3.21 ± 0.033	177	*0.083*
	$C_{3}$	none	3.26 ± 0.029	247	3.19 ± 0.052	53	*0.194*
	$A_{3}$	$B_{3}C_{3}$	3.12 ± 0.136	14	3.21 ± 0.098	286	0.323
	$B_{3}$	$A_{3}C_{3}$	3.27 ± 0.090	123	3.14 ± 0.093	177	**0.005**
	$C_{3}$	$A_{3}B_{3}$	3.28 ± 0.076	247	3.12 ± 0.089	53	**0.010**
C10:1	$A_{3}$	none	0.27 ± 0.012	14	0.28 ± 0.003	286	*0.188*
	$B_{3}$	none	0.28 ± 0.004	123	0.28 ± 0.004	177	0.366
	$C_{3}$	none	0.28 ± 0.004	247	0.27 ± 0.006	53	*0.129*
	$A_{3}$	$C_{3}$	0.27 ± 0.013	14	0.28 ± 0.005	286	0.200
	$C_{3}$	$A_{3}$	0.28 ± 0.007	247	0.27 ± 0.008	53	*0.135*
C11:0	$A_{3}$	none	0.06 ± 0.005	14	0.06 ± 0.001	286	0.469
	$B_{3}$	none	0.06 ± 0.002	123	0.06 ± 0.002	177	**0.006**
	$C_{3}$	none	0.06 ± 0.002	247	0.06 ± 0.003	53	0.465
C12:0	$A_{3}$	none	3.70 ± 0.133	14	3.95 ± 0.037	286	*0.067*
	$B_{3}$	none	3.98 ± 0.049	123	3.91 ± 0.044	177	0.215
	$C_{3}$	none	3.96 ± 0.039	247	3.85 ± 0.069	53	*0.127*
	$A_{3}$	$C_{3}$	3.69 ± 0.138	14	3.93 ± 0.055	286	*0.072*
	$C_{3}$	$A_{3}$	3.88 ± 0.116	247	3.79 ± 0.128	53	*0.141*
C12:1	$A_{3}$	none	0.08 ± 0.005	14	0.09 ± 0.001	286	**0.018**
	$B_{3}$	none	0.09 ± 0.002	123	0.09 ± 0.002	177	0.988
	$C_{3}$	none	0.09 ± 0.002	247	0.09 ± 0.003	53	*0.091*
	$A_{3}$	$C_{3}$	0.08 ± 0.005	14	0.09 ± 0.002	286	**0.020**
	$C_{3}$	$A_{3}$	0.09 ± 0.006	247	0.08 ± 0.006	53	*0.107*
C13:0 *anteiso*	$A_{3}$	none	0.04 ± 0.001	14	0.04 ± 0.000	286	0.987
	$B_{3}$	none	0.04 ± 0.000	128	0.04 ± 0.000	177	0.292
	$C_{3}$	none	0.04 ± 0.000	260	0.04 ± 0.001	53	*0.109*
C13:0 *iso*	$A_{3}$	none	0.07 ± 0.004	14	0.08 ± 0.001	286	**0.049**
	$B_{3}$	none	0.08 ± 0.002	123	0.08 ± 0.001	177	0.515
	$C_{3}$	none	0.08 ± 0.001	247	0.08 ± 0.002	53	*0.119*
	$A_{3}$	$C_{3}$	0.07 ± 0.005	14	0.08 ± 0.002	286	*0.053*
	$C_{3}$	$A_{3}$	0.08 ± 0.004	247	0.07 ± 0.005	53	*0.134*
C13:0	$A_{3}$	none	0.12 ± 0.007	14	0.12 ± 0.002	286	0.954
	$B_{3}$	none	0.12 ± 0.003	123	0.12 ± 0.002	177	**0.029**
	$C_{3}$	none	0.12 ± 0.002	247	0.12 ± 0.004	53	0.328

**Table 1:** Continued.

	Mean ± SE${}^{1}$ (g/100 g milk FA)						
Individual/	Variants	Other	Absent	n	Present	n	p
grouped		variants					
fatty acids${}^{2}$		in model					
C14:0	$A_{3}$	none	12.47 ± 0.232	14	12.48 ± 0.064	286	0.963
	$B_{3}$	none	12.54 ± 0.084	123	12.43 ± 0.076	177	0.288
	$C_{3}$	none	12.51 ± 0.068	247	12.35 ± 0.120	53	0.223
C14:1	$A_{3}$	none	0.89 ± 0.067	14	0.96 ± 0.032	286	0.285
	$B_{3}$	none	0.93 ± 0.036	123	0.97 ± 0.033	177	*0.103*
	$C_{3}$	none	0.96 ± 0.033	247	0.93 ± 0.042	53	0.353
C14:1 *cis*-9	$A_{3}$	none	0.88 ± 0.059	14	0.95 ± 0.016	286	0.221
	$B_{3}$	none	0.93 ± 0.022	123	0.97 ± 0.019	177	*0.122*
	$C_{3}$	none	0.96 ± 0.017	247	0.93 ± 0.031	53	0.368
C15:0	$A_{3}$	none	1.50 ± 0.049	14	1.48 ± 0.014	286	0.664
	$B_{3}$	none	1.50 ± 0.018	123	1.46 ± 0.016	177	*0.063*
	$C_{3}$	none	1.48 ± 0.014	247	1.47 ± 0.025	53	0.880
C15:0 *anteiso*	$A_{3}$	none	0.67 ± 0.026	14	0.64 ± 0.007	286	0.265
	$B_{3}$	none	0.64 ± 0.009	123	0.64 ± 0.009	177	0.841
	$C_{3}$	none	0.64 ± 0.008	247	0.62 ± 0.013	53	0.277
C15:1	$A_{3}$	none	0.30 ± 0.009	14	0.28 ± 0.002	286	**0.043**
	$B_{3}$	none	0.28 ± 0.003	123	0.28 ± 0.003	177	0.698
	$C_{3}$	none	0.28 ± 0.003	247	0.28 ± 0.005	53	0.370
C16:1 *cis*-9	$A_{3}$	none	1.25 ± 0.071	14	1.27 ± 0.020	286	0.792
	$B_{3}$	none	1.26 ± 0.026	123	1.27 ± 0.023	177	0.948
	$C_{3}$	none	1.26 ± 0.021	247	1.28 ± 0.037	53	0.672
C17:0 *iso*	$A_{3}$	none	0.56 ± 0.019	14	0.55 ± 0.005	286	0.464
	$B_{3}$	none	0.54 ± 0.007	123	0.56 ± 0.006	177	**0.020**
	$C_{3}$	none	0.55 ± 0.005	247	0.53 ± 0.010	53	**0.042**
	$B_{3}$	$C_{3}$	0.54 ± 0.007	123	0.56 ± 0.006	177	**0.020**
	$C_{3}$	$B_{3}$	0.55 ± 0.007	247	0.54 ± 0.011	53	*0.164*
C17:0	$A_{3}$	none	0.87 ± 0.023	14	0.87 ± 0.006	286	0.879
	$B_{3}$	none	0.88 ± 0.008	123	0.87 ± 0.008	177	*0.183*
	$C_{3}$	none	0.87 ± 0.007	247	0.88 ± 0.012	53	0.583
C17:1	$A_{3}$	none	0.20 ± 0.007	14	0.20 ± 0.002	286	0.732
	$B_{3}$	none	0.20 ± 0.003	123	0.20 ± 0.002	177	0.661
	$C_{3}$	none	0.20 ± 0.002	247	0.20 ± 0.004	53	0.728
C18:1 *trans*-5, 10	$A_{3}$	none	0.31 ± 0.012	14	0.30 ± 0.003	286	0.200
	$B_{3}$	none	0.29 ± 0.004	123	0.30 ± 0.004	177	0.779
	$C_{3}$	none	0.30 ± 0.004	247	0.30 ± 0.006	53	0.693
C18:1 *trans*-11	$A_{3}$	none	3.17 ± 0.203	14	2.73 ± 0.056	286	**0.031**
	$B_{3}$	none	2.75 ± 0.075	123	2.74 ± 0.067	177	0.897
	$C_{3}$	none	2.76 ± 0.060	247	2.70 ± 0.106	53	0.583
C18:2 *trans*-9, 12	$A_{3}$	none	0.42 ± 0.011	14	0.42 ± 0.003	286	0.921
	$B_{3}$	none	0.42 ± 0.004	123	0.41 ± 0.004	177	0.523
	$C_{3}$	none	0.41 ± 0.003	247	0.42 ± 0.006	53	0.642
C18:2 *cis*-9, *trans*-12	$A_{3}$	none	0.08 ± 0.006	14	0.07 ± 0.002	286	0.291
	$B_{3}$	none	0.07 ± 0.002	123	0.07 ± 0.002	177	0.300
	$C_{3}$	none	0.07 ± 0.002	247	0.07 ± 0.003	53	0.847

**Table 1:** Continued.

	Mean ± SE${}^{1}$ (g/100 g milk FA)						
Individual/	Variants	Other	Absent	n	Present	n	p
grouped		variants					
fatty acids${}^{2}$		in model					
C18:2 *trans*-9, *cis*-12	$A_{3}$	none	0.54 ± 0.032	14	0.47 ± 0.009	286	**0.029**
	$B_{3}$	none	0.47 ± 0.012	123	0.47 ± 0.011	177	0.628
	$C_{3}$	none	0.47 ± 0.010	247	0.47 ± 0.017	53	0.769
C18:2 *cis*-9, 12	$A_{3}$	none	0.66 ± 0.022	14	0.69 ± 0.006	286	*0.132*
	$B_{3}$	none	0.68 ± 0.008	123	0.70 ± 0.007	177	*0.055*
	$C_{3}$	none	0.70 ± 0.006	247	0.68 ± 0.011	53	0.213
	$A_{3}$	$B_{3}$	0.66 ± 0.023	14	0.69 ± 0.010	286	*0.103*
	$B_{3}$	$A_{3}$	0.67 ± 0.019	123	0.69 ± 0.018	177	**0.045**
C18:2 *cis*-9, *trans*-13	$A_{3}$	none	0.29 ± 0.010	14	0.29 ± 0.003	286	0.954
	$B_{3}$	none	0.29 ± 0.004	123	0.29 ± 0.003	177	0.971
	$C_{3}$	none	0.29 ± 0.003	247	0.29 ± 0.005	53	0.796
C18:3 *cis*-9, 12, 15	$A_{3}$	none	0.76 ± 0.030	14	0.80 ± 0.008	286	*0.154*
	$B_{3}$	none	0.79 ± 0.011	123	0.81 ± 0.010	177	*0.088*
	$C_{3}$	none	0.80 ± 0.009	247	0.79 ± 0.016	53	0.258
	$A_{3}$	$B_{3}$	0.75 ± 0.032	14	0.80 ± 0.013	286	*0.126*
	$B_{3}$	$A_{3}$	0.77 ± 0.024	123	0.80 ± 0.023	177	*0.075*
C19:0	$A_{3}$	none	0.14 ± 0.008	14	0.14 ± 0.002	286	0.353
	$B_{3}$	none	0.14 ± 0.003	123	0.14 ± 0.002	177	0.906
	$C_{3}$	none	0.14 ± 0.002	247	0.14 ± 0.004	53	0.402
C20:0	$A_{3}$	none	0.13 ± 0.005	14	0.13 ± 0.001	286	0.932
	$B_{3}$	none	0.13 ± 0.002	123	0.13 ± 0.002	177	**0.019**
	$C_{3}$	none	0.13 ± 0.001	247	0.13 ± 0.002	53	**0.027**
	$B_{3}$	$C_{3}$	0.13 ± 0.002	123	0.13 ± 0.002	177	**0.033**
	$C_{3}$	$B_{3}$	0.13 ± 0.002	247	0.13 ± 0.003	53	*0.073*
C20:1 *cis*-5	$A_{3}$	none	0.07 ± 0.004	14	0.06 ± 0.001	286	*0.099*
	$B_{3}$	none	0.06 ± 0.002	123	0.06 ± 0.001	177	0.833
	$C_{3}$	none	0.06 ± 0.001	247	0.06 ± 0.002	53	0.989
C20:1 *cis*-9	$A_{3}$	none	0.15 ± 0.007	14	0.15 ± 0.002	286	0.772
	$B_{3}$	none	0.15 ± 0.002	123	0.15 ± 0.002	177	0.644
	$C_{3}$	none	0.15 ± 0.002	247	0.16 ± 0.003	53	0.303
C20:1 *cis*-11	$A_{3}$	none	0.07 ± 0.004	14	0.08 ± 0.001	286	0.222
	$B_{3}$	none	0.08 ± 0.001	123	0.08 ± 0.001	177	0.778
	$C_{3}$	none	0.08 ± 0.001	247	0.08 ± 0.002	53	0.454
C20:3 *cis*-8, 11, 14	$A_{3}$	none	0.03 ± 0.002	14	0.03 ± 0.000	286	0.300
	$B_{3}$	none	0.03 ± 0.001	123	0.03 ± 0.001	177	0.447
	$C_{3}$	none	0.03 ± 0.000	247	0.03 ± 0.001	53	0.859
C20:4 *cis*-5, 8, 11, 14	$A_{3}$	none	0.04 ± 0.002	14	0.03 ± 0.001	286	0.269
	$B_{3}$	none	0.03 ± 0.001	123	0.03 ± 0.001	177	0.978
	$C_{3}$	none	0.04 ± 0.001	247	0.03 ± 0.001	53	0.439
C20:5 *cis*-5, 8, 11, 14, 17	$A_{3}$	none	0.09 ± 0.003	14	0.09 ± 0.001	286	*0.143*
	$B_{3}$	none	0.09 ± 0.001	123	0.09 ± 0.001	177	0.893
	$C_{3}$	none	0.09 ± 0.001	247	0.09 ± 0.002	53	0.993
C22:0	$A_{3}$	none	0.08 ± 0.004	14	0.07 ± 0.001	286	**0.003**
	$B_{3}$	none	0.06 ± 0.001	123	0.07 ± 0.001	177	**0.035**
	$C_{3}$	none	0.07 ± 0.001	247	0.07 ± 0.002	53	0.666
	$A_{3}$	B${}_{3}$	0.08 ± 0.004	14	0.06 ± 0.001	286	**0.003**
	$B_{3}$	A${}_{3}$	0.07 ± 0.005	123	0.07 ± 0.005	177	*0.053*

**Table 1:** Continued.

	Mean ± SE${}^{1}$ (g/100 g milk FA)						
Individual/	Variants	Other	Absent	n	Present	n	p
grouped		variants					
fatty acids${}^{2}$		in model					
C22:1 *trans*-13	$A_{3}$	none	0.07 ± 0.004	14	0.07 ± 0.001	286	0.355
	$B_{3}$	none	0.07 ± 0.001	123	0.07 ± 0.001	177	0.642
	$C_{3}$	none	0.07 ± 0.001	247	0.07 ± 0.002	53	*0.148*
C24:0	$A_{3}$	none	0.05 ± 0.002	14	0.04 ± 0.001	286	**0.006**
	$B_{3}$	none	0.04 ± 0.001	123	0.05 ± 0.001	177	**0.021**
	$C_{3}$	none	0.04 ± 0.001	247	0.05 ± 0.001	53	0.891
	$A_{3}$	$B_{3}$	0.05 ± 0.003	14	0.04 ± 0.001	286	**0.008**
	$B_{3}$	$A_{3}$	0.05 ± 0.003	123	0.05 ± 0.003	177	**0.031**
C22:5 *cis*-7, 10, 13, 16, 19	$A_{3}$	none	0.13 ± 0.007	14	0.12 ± 0.002	286	0.315
	$B_{3}$	none	0.12 ± 0.002	123	0.12 ± 0.002	177	0.877
	$C_{3}$	none	0.12 ± 0.002	247	0.12 ± 0.003	53	0.213
SCFA	$A_{3}$	none	2.84 ± 0.063	14	2.82 ± 0.017	286	0.800
	$B_{3}$	none	2.84 ± 0.023	123	2.81 ± 0.021	177	0.177
	$C_{3}$	none	2.82 ± 0.018	247	2.83 ± 0.044	53	0.674
MCFA	$A_{3}$	none	20.46 ± 0.444	14	20.85 ± 0.123	286	0.369
	$B_{3}$	none	20.99 ± 0.161	123	20.73 ± 0.146	177	*0.151*
	$C_{3}$	none	20.91 ± 0.131	247	20.56 ± 0.230	53	*0.152*
	$B_{3}$	$C_{3}$	20.95 ± 0.393	123	20.40 ± 0.408	177	**0.011**
	$C_{3}$	$B_{3}$	21.01 ± 0.308	247	20.30 ± 0.373	53	**0.011**
LCFA	$A_{3}$	none	48.75 ± 0.739	14	48.93 ± 0.205	286	0.802
	$B_{3}$	none	48.94 ± 0.269	123	48.92 ± 0.243	177	0.938
	$C_{3}$	none	48.82 ± 0.217	247	49.37 ± 0.382	53	*0.171*
MUFA	$A_{3}$	none	20.36 ± 0.512	14	19.98 ± 0.142	286	0.457
	$B_{3}$	none	19.82 ± 0.186	123	20.12 ± 0.168	177	*0.170*
	$C_{3}$	none	20.00 ± 0.151	247	19.94 ± 0.265	53	0.829
PUFA	$A_{3}$	none	4.25 ± 0.132	14	4.08 ± 0.037	286	0.209
	$B_{3}$	none	4.07 ± 0.048	123	4.10 ± 0.044	177	0.475
	$C_{3}$	none	4.10 ± 0.039	247	4.03 ± 0.068	53	0.300
C18:1 all *trans*	$A_{3}$	none	3.48 ± 0.207	14	3.03 ± 0.058	286	**0.029**
	$B_{3}$	none	3.05 ± 0.076	123	3.048 ± 0.069	177	0.912
	$C_{3}$	none	3.05 ± 0.062	247	2.10 ± 0.108	53	0.607
all C18:3	$A_{3}$	none	0.83 ± 0.031	14	0.88 ± 0.009	286	*0.165*
	$B_{3}$	none	0.86 ± 0.011	123	0.88 ± 0.010	177	*0.091*
	$C_{3}$	none	0.88 ± 0.009	247	0.86 ± 0.016	53	0.277
	$A_{3}$	$B_{3}$	0.83 ± 0.033	14	0.87 ± 0.013	286	*0.136*
	$B_{3}$	$A_{3}$	0.85 ± 0.024	123	0.87 ± 0.023	177	*0.079*
Omega 3	$A_{3}$	none	0.10 ± 0.031	14	1.02 ± 0.009	286	0.353
	$B_{3}$	none	1.01 ± 0.011	123	1.03 ± 0.010	177	*0.083*
	$C_{3}$	none	1.03 ± 0.009	247	1.00 ± 0.016	53	*0.173*
	$B_{3}$	$C_{3}$	1.01 ± 0.011	123	1.03 ± 0.010	177	*0.083*
	$C_{3}$	$B_{3}$	1.03 ± 0.011	247	1.00 ± 0.017	53	0.281
Omega 6	$A_{3}$	none	0.80 ± 0.023	14	0.83 ± 0.007	286	0.211
	$B_{3}$	none	0.82 ± 0.009	123	0.84 ± 0.008	177	*0.059*
	$C_{3}$	none	0.83 ± 0.007	247	0.82 ± 0.012	53	0.223
	$A_{3}$	$B_{3}$	0.80 ± 0.025	14	0.83 ± 0.011	286	*0.170*
	$B_{3}$	$A_{3}$	0.81 ± 0.016	123	0.83 ± 0.016	177	*0.052*

**Table 1:** Continued.

	Mean ± SE${}^{1}$ (g/100 g milk FA)						
Individual/	Variants	Other	Absent	n	Present	n	p
grouped		variants					
fatty acids${}^{2}$		in model					
C10:1 index	$A_{3}$	none	7.85 ± 0.384	14	8.05 ± 0.107	286	0.591
	$B_{3}$	none	7.87 ± 0.139	123	8.18 ± 0.126	177	*0.052*
	$C_{3}$	none	8.07 ± 0.113	247	7.95 ± 0.199	53	0.550
C12:1 index	$A_{3}$	none	2.07 ± 0.099	14	2.25 ± 0.028	286	*0.074*
	$B_{3}$	none	2.21 ± 0.036	123	2.26 ± 0.033	177	0.227
	$C_{3}$	none	2.25 ± 0.029	247	2.19 ± 0.052	53	0.243
C14:1 index	$A_{3}$	none	6.60 ± 0.434	14	7.11 ± 0.120	286	0.236
	$B_{3}$	None	6.92 ± 0.158	123	7.23 ± 0.143	177	*0.081*
	$C_{3}$	none	7.12 ± 0.128	247	6.10 ± 0.225	53	0.598
C16:1 index	$A_{3}$	none	3.24 ± 0.160	14	3.26 ± 0.044	286	0.926
	$B_{3}$	None	3.26 ± 0.058	123	3.26 ± 0.053	177	0.962
	$C_{3}$	none	3.26 ± 0.047	247	3.27 ± 0.083	53	0.880
CLA *cis*-9, *trans*-11	$A_{3}$	none	1.14 ± 0.085	14	0.99 ± 0.024	286	*0.090*
	$B_{3}$	none	0.99 ± 0.031	123	1.00 ± 0.028	177	0.762
	$C_{3}$	none	1.00 ± 0.025	247	0.97 ± 0.044	53	0.484

${}^{1}$ Predicted means and standard error of those means derived from general linear mixed-effects models (GLMMs). Cow age (categorical variable), *LEP* variants (categorical variable), herd (categorical variable) and days in milk (continuous variable) were fitted to the model as fixed effects. 0.05<p<0.2 in italics, p<0.05 in bold. ${}^{2}$ SCFA – short-chain fatty acid; MCFA – medium-chain fatty acid; LCFA – long-chain fatty acid; MUFA – monounsaturated fatty acid; PUFA – polyunsaturated fatty acid; UFA – unsaturated fatty acid; SFA – saturated fatty acid. The unit (g/100 g milk FA) applied to all FAs except for the FA indices which had a unit of %.

### 3.3 Genotype models

Only the genotypes $A_{3}A_{3}$ (n = 70), $A_{3}B_{3}$ (n = 166) and $A_{3}C_{3}$ (n = 50) with a frequency greater than 5 % were analysed. The other genotypes $B_{3}B_{3}$ (n = 11) and $C_{3}C_{3}$ (n = 3) were not included in this model. The composition of milk fat was affected by genotype, and the results were consistent with the findings of the variant presence/absence models. Cows carrying the $A_{3}A_{3}$ (most common) genotype contained higher levels of saturated fatty acids (SFAs), but when one copy of the $A_{3}$ variant was replaced by $B_{3}$ or $C_{3}$, the resulting heterozygous genotype ($A_{3}B_{3}$ or $A_{3}C_{3}$) was associated with changed levels of SFAs in milk. Cows carrying the $A_{3}B_{3}$ genotype was associated with increased levels of C22:0 and C24:0 but decreased C8:0, C10:0, C11:0, C13:0, C15:0 and grouped MCFA levels (p<0.05). $A_{3}C_{3}$ was found to be associated with decreased levels of C10:0, C11:0, C13:0 and grouped MCFA (p<0.05; Table 2).

**Table 2:** Associations between milk fatty acid levels and leptin genotypes.

	Mean ± SE${}^{1}$ (g/100 g milk FA)			p
	$A_{3}A_{3}$	$A_{3}B_{3}$	$A_{3}C_{3}$	
Individual/grouped fatty acids${}^{2}$	n = 70	n = 166	n = 50	
C4:0	1.27 ± 0.016	1.27 ± 0.012	1.27 ± 0.019	0.942
C6:0	1.58 ± 0.015	1.55 ± 0.011	1.55 ± 0.017	0.055
C8:0	1.21 ± 0.012${}^{a}$	1.17 ± 0.009${}^{b}$	1.17 ± 0.014${}^{\mathrm{ab}}$	**0.013**
C10:0	3.36 ± 0.046${}^{a}$	3.21 ± 0.034${}^{b}$	3.19 ± 0.053${}^{b}$	**0.009**
C10:1	0.28 ± 0.006	0.28 ± 0.004	0.28 ± 0.006	0.480
C11:0	0.07 ± 0.002${}^{a}$	0.06 ± 0.002${}^{b}$	0.06 ± 0.003${}^{b}$	**0.001**
C12:1	0.09 ± 0.002	0.09 ± 0.002	0.09 ± 0.003	0.277
C13:0 *iso*	0.08 ± 0.002	0.08 ± 0.002	0.08 ± 0.002	0.491
C13:0 *anteiso*	0.04 ± 0.001	0.04 ± 0.000	0.04 ± 0.001	0.295
C13:0	0.13 ± 0.003${}^{a}$	0.12 ± 0.002${}^{b}$	0.12 ± 0.004${}^{b}$	**0.002**
C14:0	12.67 ± 0.107	12.42 ± 0.078	12.37 ± 0.123	0.061
C14:1 *cis*-9	0.93 ± 0.027	0.97 ± 0.020	0.94 ± 0.032	0.325
C15:0	1.52 ± 0.023${}^{a}$	1.45 ± 0.016${}^{b}$	1.48 ± 0.026${}^{\mathrm{ab}}$	**0.040**
C15:1	0.29 ± 0.004	0.28 ± 0.003	0.28 ± 0.005	0.670
C16:0	37.28 ± 0.383	37.42 ± 0.279	37.74 ± 0.439	0.532
C16:1 *cis*-9	1.25 ± 0.033	1.26 ± 0.024	1.29 ± 0.038	0.724
C17:0 *iso*	0.55 ± 0.009	0.56 ± 0.006	0.54 ± 0.010	0.124
C17:0	0.88 ± 0.011	0.87 ± 0.008	0.88 ± 0.012	0.418
C18:1 *trans*-11	2.82 ± 0.094	2.73 ± 0.069	2.71 ± 0.108	0.608
C18:2 *trans*-9, 12	0.42 ± 0.005	0.41 ± 0.004	0.42 ± 0.006	0.885
C18:2 *cis*-9, *trans*-13	0.29 ± 0.004	0.29 ± 0.003	0.29 ± 0.005	0.955
C18:2 *cis*-9, *trans*-12	0.07 ± 0.003	0.07 ± 0.002	0.07 ± 0.003	0.413
C18:2 *trans*-9, *cis*-12	0.48 ± 0.015	0.46 ± 0.011	0.47 ± 0.017	0.494
C18:2 *cis*-9, 12	0.68 ± 0.010	0.70 ± 0.007	0.68 ± 0.012	0.103
C18:3 *cis*-6, 9, 12	0.07 ± 0.001	0.07 ± 0.001	0.08 ± 0.002	0.839
C18:3* cis*-9, 12, 15	0.79 ± 0.014	0.81 ± 0.010	0.78 ± 0.016	0.128
C19:0	0.15 ± 0.004	0.14 ± 0.003	0.14 ± 0.004	0.603
C20:0	0.13 ± 0.002	0.13 ± 0.002	0.13 ± 0.003	0.055
C20:1 *cis*-5	0.06 ± 0.002	0.06 ± 0.001	0.06 ± 0.002	0.854
C20:1 *cis*-9	0.15 ± 0.003	0.15 ± 0.002	0.15 ± 0.004	0.625
C20:1 *cis*-11	0.08 ± 0.002	0.08 ± 0.001	0.08 ± 0.002	0.784
C20:4 *cis*-5, 8, 11, 14	0.04 ± 0.001	0.03 ± 0.001	0.03 ± 0.001	0.641
C22:0	0.06 ± 0.002${}^{a}$	0.07 ± 0.001${}^{b}$	0.07 ± 0.003${}^{\mathrm{ab}}$	**0.032**
C24:0	0.04 ± 0.001${}^{a}$	0.05 ± 0.001${}^{b}$	0.04 ± 0.001${}^{\mathrm{ab}}$	**0.026**
C22:5 *cis*-7, 10, 13, 16, 19	0.12 ± 0.003	0.12 ± 0.002	0.12 ± 0.003	0.289
SCFA	2.85 ± 0.029	2.81 ± 0.021	2.82 ± 0.034	0.437
MCFA	21.31 ± 0.205${}^{a}$	20.72 ± 0.149${}^{b}$	20.60 ± 0.235${}^{b}$	**0.015**
LCFA	48.55 ± 0.337	48.91 ± 0.246	49.25 ± 0.387	0.343
MUFA	19.78 ± 0.234	20.14 ± 0.171	20.01 ± 0.269	0.388
PUFA	4.11 ± 0.061	4.10 ± 0.044	4.04 ± 0.070	0.656
C18:1 all *trans*	3.11 ± 0.097	3.02 ± 0.070	3.00 ± 0.111	0.640
all C18:3	0.86 ± 0.015	0.89 ± 0.011	0.86 ± 0.017	0.143
Omega 3	1.02 ± 0.015	1.04 ± 0.011	1.00 ± 0.017	0.106
Omega 6	0.82 ± 0.011	0.84 ± 0.008	0.82 ± 0.013	0.126
branched FA	1.60 ± 0.019	1.60 ± 0.014	1.57 ± 0.022	0.259
Total C18:2	2.96 ± 0.058	2.93 ± 0.042	2.90 ± 0.066	0.777
Total C18:3	0.86 ± 0.015	0.89 ± 0.011	0.86 ± 0.017	0.144
Total UFA	23.89 ± 0.280	24.24 ± 0.204	24.05 ± 0.321	0.506
Total SFA	68.84 ± 0.304	68.70 ± 0.221	68.98 ± 0.349	0.738
unsaturated index	25.77 ± 0.305	26.09 ± 0.222	25.86 ± 0.350	0.597
C10:1 index	7.80 ± 0.179	8.19 ± 0.130	7.99 ± 0.205	0.136
C12:1 index	2.23 ± 0.046	2.27 ± 0.033	2.21 ± 0.053	0.533
C14:1 index	6.84 ± 0.201	7.26 ± 0.147	7.05 ± 0.231	0.165
C16:1 index	3.25 ± 0.074	3.25 ± 0.054	3.30 ± 0.085	0.880
C18:1 index	59.78 ± 0.463	60.08 ± 0.338	59.88 ± 0.532	0.819
CLA *cis*-9, *trans*-11	1.01 ± 0.040	0.99 ± 0.029	0.98 ± 0.045	0.819

${}^{1}$ Predicted means and standard error of those means derived from general linear mixed-effects models (GLMMs). Cow age (categorical variable), leptin genotypes (categorical variable), herd (categorical variable) and days in milk (continuous variable) were fitted to the model as fixed effects. Means within a row that do not share a superscript letter (a or b) are separated by Bonferroni test at p<0.05. 0.05<p<0.02 in italics, while p<0.05 is in bold. ${}^{2}$ SCFA – short-chain fatty acid; MCFA – medium-chain fatty acid; LCFA – long-chain fatty acid; MUFA – monounsaturated fatty acid; PUFA – polyunsaturated fatty acid; UFA – unsaturated fatty acid; SFA – saturated fatty acid. The unit (g/100 g milk FA) applied to all FAs except for the FA indices which had a unit of %.

## 4 Discussion

This is the first study investigating the effect of leptin gene variations in exon 3 with composition of milk FA in NZ HF × J cows farmed wholly outdoors on pasture.

Overall, the results presented here revealed associations between variation in the leptin gene and the composition of milk fat. Cows carrying the $A_{3}A_{3}$ genotype had higher levels of SFAs, but when one copy of the $A_{3}$ variant is replaced by a $B_{3}$ variant, the resulting heterozygous genotype $A_{3}B_{3}$ had decreased levels of SFA.

In an analysis of the effect of *LEP* nucleotide sequence variation on the FA profile of cattle muscle fat, Orrù et al. (2011) investigated the effect of c.239C/T (p.Ala80Val – also identified in this study) in 103 Simmental bulls. They revealed that the C allele (the allele with alanine at amino acid 80 – equivalent to the $A_{3}$ variant here) was associated with increased meat C14:1 and C14 index. In contrast, our study revealed the presence of variant $A_{3}$ was associated with increased C12:1 but decreased C15:1 and C18:1 all *trans*. In addition, the $B_{3}$ variant identified in this study, which carries the T in the nucleotide substitution c.239C/T (has a valine residue at position 80), was associated with a decrease in some short- and medium-chain SFAs. Taken together, the observation that the C and T alleles of c.239C/T appeared to affect the composition of FAs in meat and milk differently suggests further investigation of this substitution and its effects is required.

In another study (Avondo et al., 2019), the effects of variation in a *LEP* intron 1 microsatellite sequence and its interaction with milk FA composition, diet, milk traits, and metabolic state in Girgentana lactating goats at mid-lactation were investigated. It was revealed that the composition of milk FA was strongly influenced by *LEP* genotype. Goats with the homozygous genotype 266 bp/266 bp (L genotype) had lower levels of SFA but increased levels of MUFA and PUFA, compared to goats with the heterozygous genotype 266 bp/264 bp (H genotype). Although our results also showed a decrease in the levels of SFA, it is difficult to specifically link our results to the work of Avondo et al. (2019) because of the differences in the gene regions studied and the species investigated. In the Avondo et al. (2019) study, the differences described between the *LEP* genotypes suggested that the L genotype could be associated with a higher utilization of body fat reserves. This is consistent with the finding of higher levels of MUFA and PUFA and lower levels of SFA found with the increased mobilization of FAs from adipose tissue in other studies (Palmquist et al., 1993; Vrankovic et al., 2017). It may also be consistent with the hypothesis of increased demand for energy as reported by Di Gregorio et al. (2014) for the L genotype.

The leptin gene from both cattle and goats map to chromosome 4, and on that chromosome there are quantitative trait loci (QTLs) for fat yield and percentage in milk (Cattle QTL database https://www.animalgenome.org/cgi-in/QTLdb/BT/index, last access: 10 July 2020) and FA composition (Li et al., 2014). This suggests it would be worthwhile undertaking further research into the role of bovine *LEP* and variation in the gene in the mobilization and utilization of body fat reserves.

These previous reports, along with the findings we report, appear to contradict the findings of Marchitelli et al. (2013). Their study did not reveal any association between the p.Arg25Cys SNP in *LEP* exon 2 and milk FA traits in Jersey, Piedmontese and Valdostana cattle breeds. A number of factors may have been responsible for this disparity in findings, including the obvious difference in gene region examined and the potential effect of breed differences. While Marchitelli et al. (2013) investigated the effect of the exon 2 region carrying the non-synonymous p.Arg25Cys SNP on milk FA traits, our study examined the effect of exon 3 carrying the non-synonymous p.Ala80Val SNP. Even though both nucleotide sequence variations are non-synonymous, it is likely that these SNPs will affect the concentration of milk FAs differently, since they are located on different parts of the gene. Also, while we investigated 300 NZ cross-bred HF × J cows (albeit of no fixed breed proportion), Marchitelli et al. (2013) investigated 90 cows in total which included the Italian Piedmontese, Valdostana and Jersey breeds. These breeds differ in terms of milk-related traits, especially in the composition of milk FAs. For example, milk from Jersey cows contains higher concentrations of some short- and medium-chain SFA but lower concentrations of some UFA (Arnould and Soyeurt, 2009). Other studies have also suggested that breed is an important factor that affects milk FA content (Karijord et al., 1982; Lawless et al., 1999). It therefore seems plausible that differences in breed may underlie the discrepancies in findings.

Another possible reason for the differences in findings can be attributed to diet. In our investigation, the NZ HF × J dairy cows were all grazed on pasture (a mixture of perennial ryegrass and white clover), whereas the cows chosen by Marchitelli et al. (2013) were fed with “unifeed” (corn silage and concentrates). The pasture-based production system increases the amount of PUFA and conjugated linoleic acids (CLAs) in the milk as suggested by Chilliard et al. (2001) and Dewhurst et al. (2006). In this context, differences in diet may have contributed to the disparity between our findings and those of Marchitelli et al. (2013), especially considering a previous report that suggested diet may affect the production of milk fat (Stelwagen, 2011).

## 5 Conclusions

The findings here suggest that cows carrying the variant leptin genotype $A_{3}B_{3}$ (where the $B_{3}$ variant in exon 3 with accession number MN119554 carries the p.Ala80Val SNP) are associated with decreased SFA levels in milk. Since heterozygous cows $A_{3}B_{3}$ had reduced SFA levels, cows with the $B_{3}B_{3}$ genotype might therefore have much lower levels of SFA in their milk. Unfortunately, since there were insufficient cattle with the homozygous genotypes $B_{3}B_{3}$ in the cattle investigated, further studies involving larger sample sizes across different farms and breeds are needed to validate this claim.

## Data Availability

The original data are available upon request to the corresponding author.

## References

[bib1.bib1] Arnould VMR, Soyeurt H (2009). Genetic variability of milk fatty acids. J Appl Genet.

[bib1.bib2] Avondo M, Trana AD, Valenti B, Criscione A, Bordonaro S, Angelis AD, Giorgio D, Gregorio PD (2019). Leptin Gene Polymorphism in Goats Fed with Diet at Different Energy Levels: Effects on Feed Intake, Milk Traits, Milk Fatty Acids Composition, and Metabolic State. Anim.

[bib1.bib3] Byun SO, Fang Q, Zhou H, Hickford JGH (2009). An effective method for silver-staining DNA in large numbers of polyacrylamide gels. Anal Biochem.

[bib1.bib4] Chilliard Y, Ferlay A, Doreau M (2001). Effect of different types of forages, animal fat or marine oils in cow's diet on milk fat secretion and composition, especially conjugated linoleic acid (CLA) and polyunsaturated fatty acids. Livest Prod Sci.

[bib1.bib5] Dewhurst RJ, Shingfield KJ, Lee MRF, Scollan ND (2006). Increasing the concentrations of beneficial polyunsaturated fatty acids in milk produced by dairy cows in high-forage systems. Anim Feed Sci Technol.

[bib1.bib6] De Matteis G, Scatà MC, Grandoni F, Petrera F, Abeni F, Catillo G, Napolitano F, Moioli B (2012). Association analyses of single nucleotide polymorphisms in the leptin and leptin receptor genes on milk and morphological traits in Holstein cows. Open J Anim Sci.

[bib1.bib7] Di Gregorio P, Di Trana A, Celi P, Claps S, Rando A (2014). Comparison of goat, sheep, cattle and water buffalo leptin (*LEP*) genes and effects of the Intron 1 microsatellite polymorphism in goats. Anim Prod Sci.

[bib1.bib8] Feuermann Y, Mabjeesh SJ, Shamay A (2004). Leptin affects prolactin action on milk protein and fat synthesis in the bovine mammary gland. J Dairy Sci.

[bib1.bib9] Giblin L, Butler ST, Kearney BM, Waters SM, Callanan MJ, Berry DP (2010). Association of bovine leptin polymorphisms with energy output and energy storage traits in progeny tested Holstein-Friesian dairy cattle sires. BMC Genet.

[bib1.bib10] Gong H, Zhou H, Hickford JGH (2011). Diversity of the glycine/tyrosine-rich keratin-associated protein 6 gene (KAP6) family in sheep. Mol Biol Rep.

[bib1.bib11] Hajihosseinlo A, Hashemi A, Sadeghi S (2012). Association between polymorphism in exon 3 of leptin gene and growth traits in the Makooei sheep of Iran. Livest Res Rural Dev.

[bib1.bib12] Halaas JL, Gajiwala KS, Maffei M, Cohen SL, Chait BT, Rabinowitz D, Lallone RL, Burley SK, Friedman JM (1995). Weight-reducing effects of the plasma protein encoded by the obese gene. Science.

[bib1.bib13] Haruna IL, Hadebe SA, Oladosu OJ, Mahmoud G, Zhou H, Hickford GHJ (2020). Identification of novel nucleotide sequence variations in an extended region of the bovine leptin gene (*LEP*) across a variety of cattle breeds from New Zealand and Nigeria. Arch Anim Breed.

[bib1.bib14] Houseknecht KL, Baile CA, Matteri RL, Spurlock ME (1998). The biology of leptin: A review. J Anim Sci.

[bib1.bib15] Karijord O, Standal N, Syrstad O (1982). Sources of variation in composition of milk fat. Z Tierz Zuchtungsbio.

[bib1.bib16] Kawaguchi F, Okura K, Oyama K, Mannen H, Sasazaki S (2017). Identification of leptin gene polymorphisms associated with carcass traits and fatty acid composition in Japanese Black cattle. Anim Sci J.

[bib1.bib17] Lagonigro R, Wiener P, Pilla F, Woolliams JA, Williams JL (2003). Short Communication: A new mutation in the coding region of the bovine leptin gene associated with feed intake. Anim Genet.

[bib1.bib18] Lawless F, Stanton C, Escop P, Devery R, Dillon P, Murphy JJ (1999). Influence of breed on bovine milk *cis*-9,*trans*-11-conjugated linoleic acid content. Livest Prod Sci.

[bib1.bib19] Li C, Sun D, Zhang S, Wang S, Wu X, Zhang Q, Liu L, Li Y, Qiao L (2014). Genome Wide Association Study Identifies 20 Novel Promising Genes Associated with Milk Fatty Acid Traits in Chinese Holstein. PLoS ONE.

[bib1.bib20] Li Y, Zhou H, Cheng L, Edwards GR, Hickford JGH (2019). Effect of DGAT1 variant (K232A) on milk traits and milk fat composition in outdoor pasture-grazed dairy cattle. New Zeal J Agr Res.

[bib1.bib21] Liefers SC, te Pas MFW, Veerkamp RF, van der Lende T (2002). Associations between leptin gene polymorphisms and production, live weight, energy balance, feed intake, and fertility in Holstein heifers. J Dairy Sci.

[bib1.bib22] Marchitelli C, Contarini G, De Matteis G, Crisa A, Pariset L, Scata MC, Catillo G, Napolitano F, Moioli B (2013). Milk fatty acid variability: Effect of some candidate genes involved in lipid synthesis. J Dairy Res.

[bib1.bib23] Orrù L, Cifuni GF, Piasentier E, Corazzin M, Bovolenta S, Moioli B (2011). Association analyses of single nucleotide polymorphisms in the *LEP* and *SCDI* genes on the fatty acid profile of muscle fat in Simmental bulls. Meat Sci.

[bib1.bib24] Palmquist DL, Beaulieu AD, Barbano DM (1993). Feed and Animal Factors Influencing Milk Fat Composition. J Dairy Sci.

[bib1.bib25] Pegolo S, Cecchinato A, Mele M, Conte G, Schiavon S, Bittante G (2016). Effect of candidate gene polymorphisms on the detailed fatty acids profile determined by gas chromatography in bovine milk. J Dairy Sci.

[bib1.bib26] Pomp D, Zou T, Clutter AC, Barendse W (1997). Rapid communication: mapping of leptin to bovine chromosome 4 by linkage analysis of a PCR-based polymorphism. J Anim Sci.

[bib1.bib27] Stelwagen K, Fuquay JW (2016). Mammary Gland, Milk Biosynthesis and Secretion Lactose. Encyclopedia of Dairy Science.

[bib1.bib28] Tang-Christensen M, Havel PJ, Jacobs RR, Larsen PJ, Cameron JL (1999). Central administration of leptin inhibits food intake and activates the sympathetic nervous system in rhesus macaques. J Clin Endocrinol Metab.

[bib1.bib29] Vrankovic L, Aladrovic J, Octenjak D, Bijelic D, Cvetnic L, Stojevic Z (2017). Milk fatty acid composition as an indicator of energy status in Holstein dairy cows. Arch Anim Breed.

[bib1.bib30] Zhou H, Hickford JGH, Fang Q (2006). A two-step procedure for extracting genomic DNA from dried blood spots on filter paper for polymerase chain reaction amplification. Anal Biochem.

